# Supplementary figures and images for: Sestrin3 confers resistance to recombinant human arginase in small cell lung cancer by activating Akt/mTOR/ASS1 axis
Source: PLoS One. 2025 Dec 29;20(12):e0338802. doi: 10.1371/journal.pone.0338802 (PMC12747410; doi:10.1371/journal.pone.0338802)

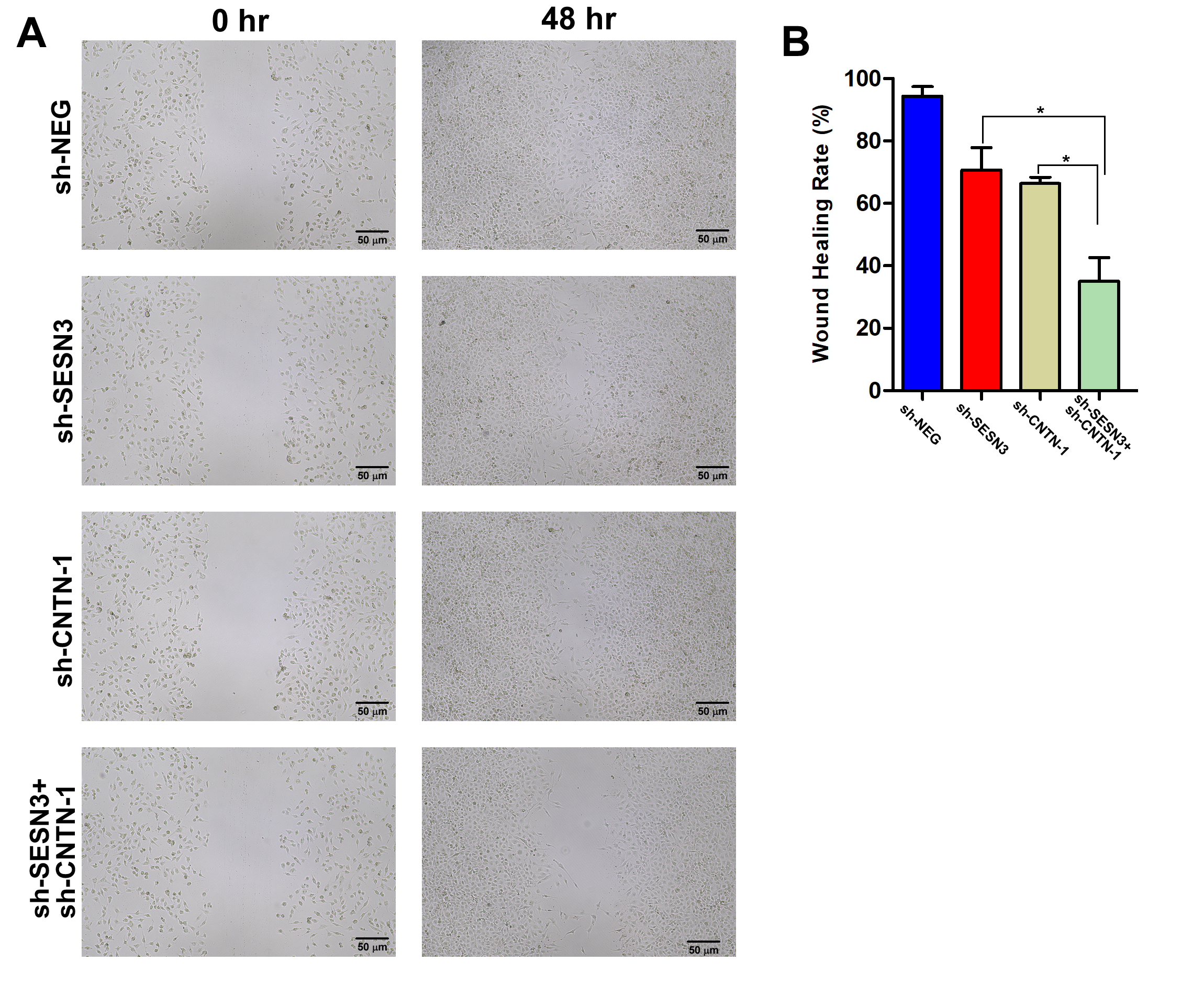

Supplement: S1 Fig — (A) Wound healing assay to assess cellular migration ability of H446-BR infected with shNEG, shSENS3, shCNTN-1, and shSESN3 plus shCNTN-1. (B) Quantitation of migration rate 48 hr in each group. (TIF) [file pone.0338802.s001.tif]

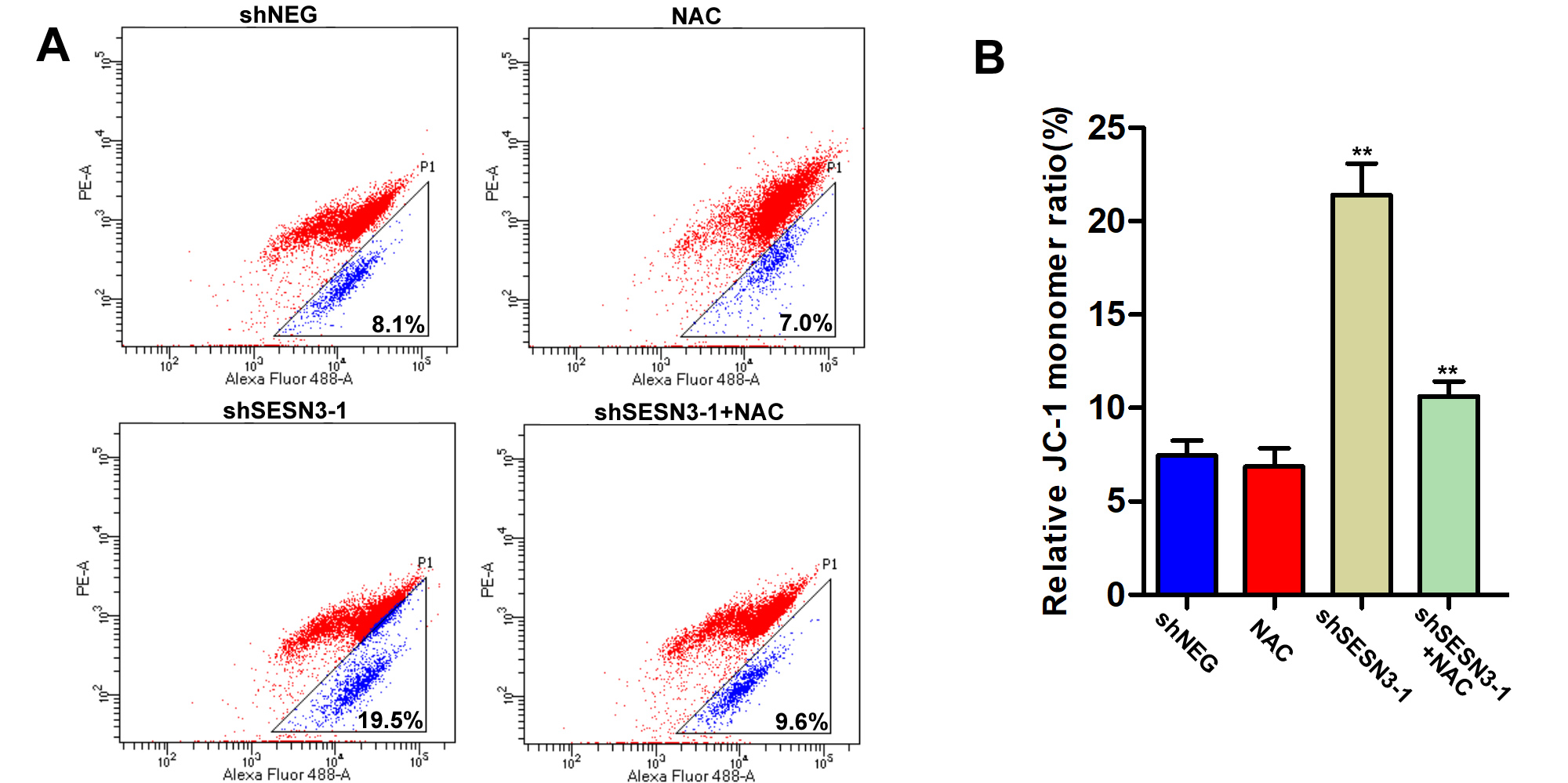

Supplement: S2 Fig — (A) Mitochondrial membrane depolarization shown by JC-1 staining in H446-BR cells (shNEG, NAC(5nM), shSESN3−1, shSESN3−1 + NAC) upon BCT-100 (20 mU/ml) exposure for 24 hr. (B) Quantitation of relative JC-1 monomer ratio in each group. (TIF) [file pone.0338802.s002.tif]

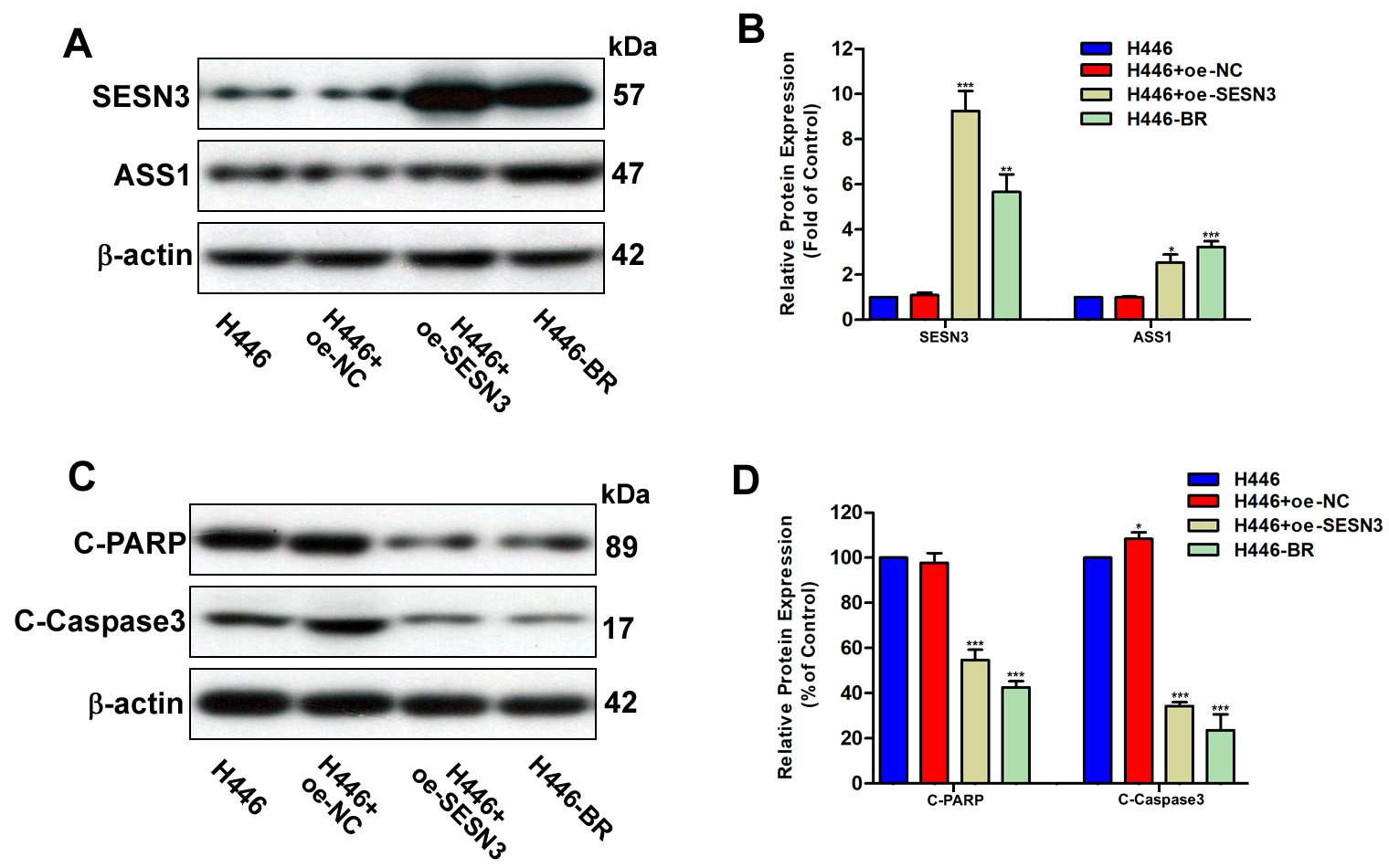

Supplement: S3 Fig — (A)Western blot to evaluate the level of ASS1 and SESN3 in H446, H446 overexpressed with normal control, H446 overexpressed with SESN3, and H446-BR. (B) Quantitation of SESN3 and ASS1 in each group. (C) Western blot to evaluate the level of C-PARP and C-Caspase3 in presence of BCT-100 treatment (20 mU/ml, 72 hr). (D) Quantitation of C-PARP and C-Caspase3 in each group. β-Actin was used as loading control. Error bars indicate the mean (SD); n = 3. P-values were determined using one-way ANOVA (*P < 0.05, **P < 0.01, ***P < 0.001). (TIF) [file pone.0338802.s003.tif]

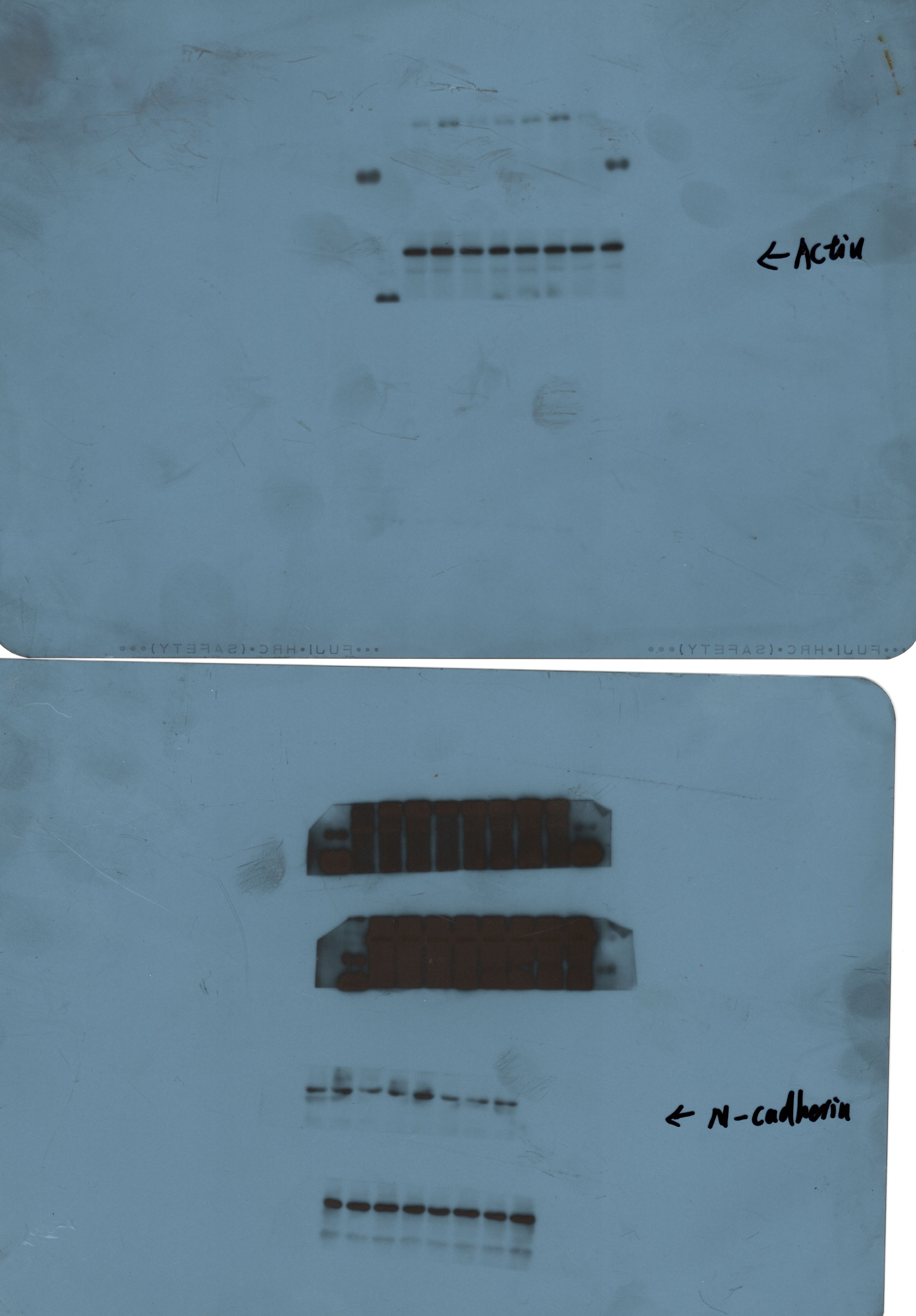

Supplement: S1 File — (ZIP) [file pone.0338802.s006.zip › WB Raw data/Fig 3f-1.jpg]

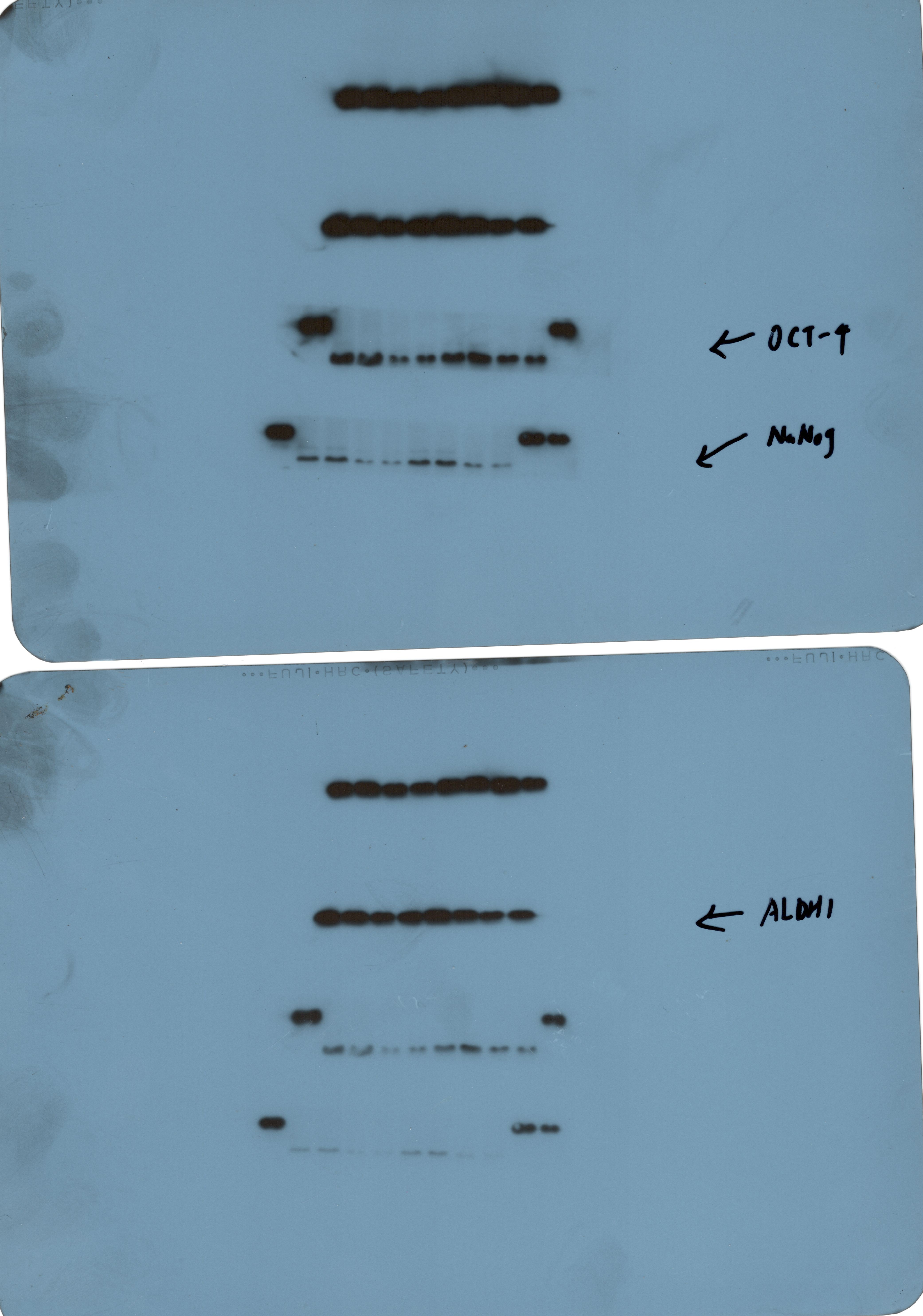

Supplement: S1 File — (ZIP) [file pone.0338802.s006.zip › WB Raw data/Fig 3f-2.jpg]

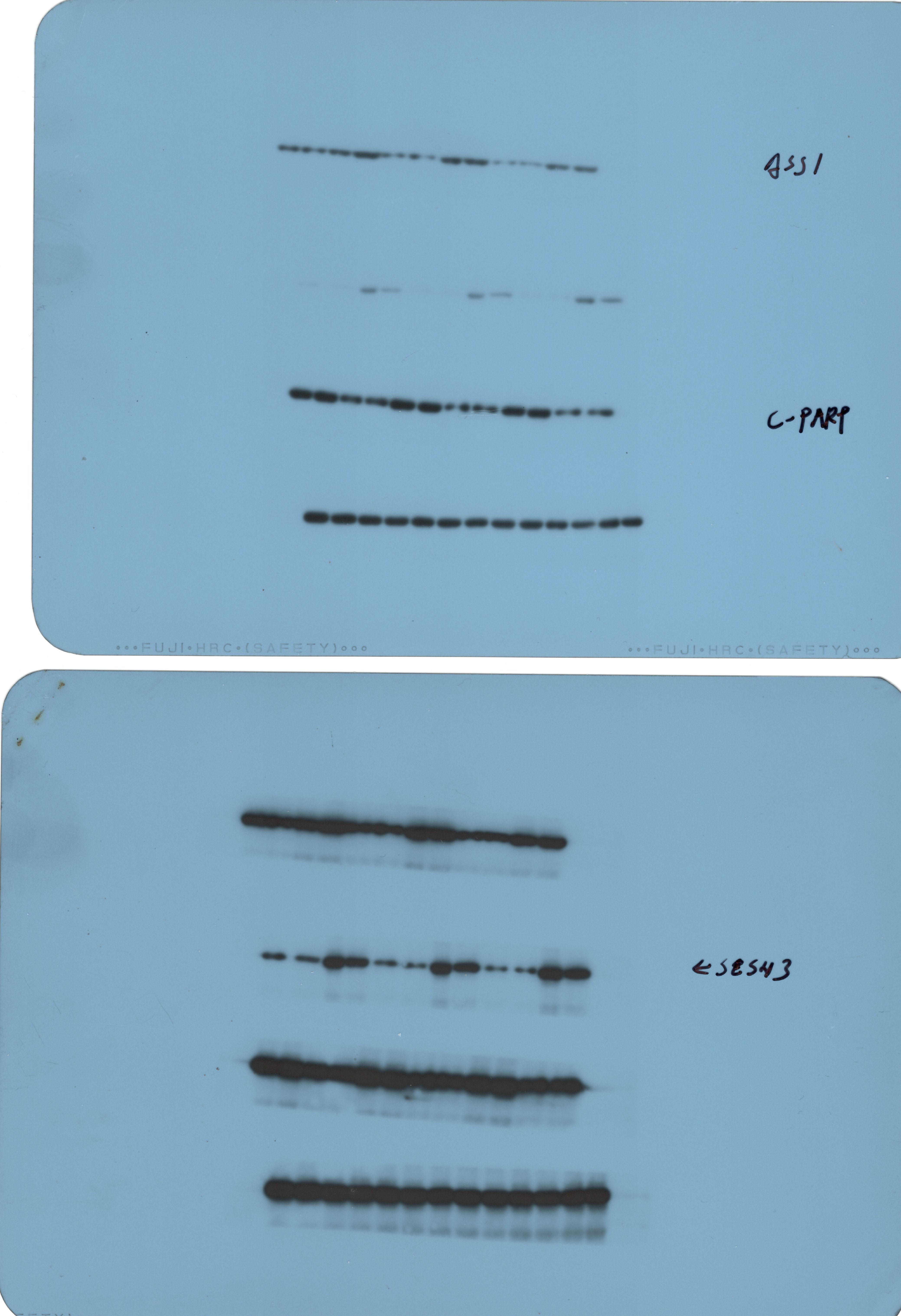

Supplement: S1 File — (ZIP) [file pone.0338802.s006.zip › WB Raw data/Fig S3-1 (1).jpg]

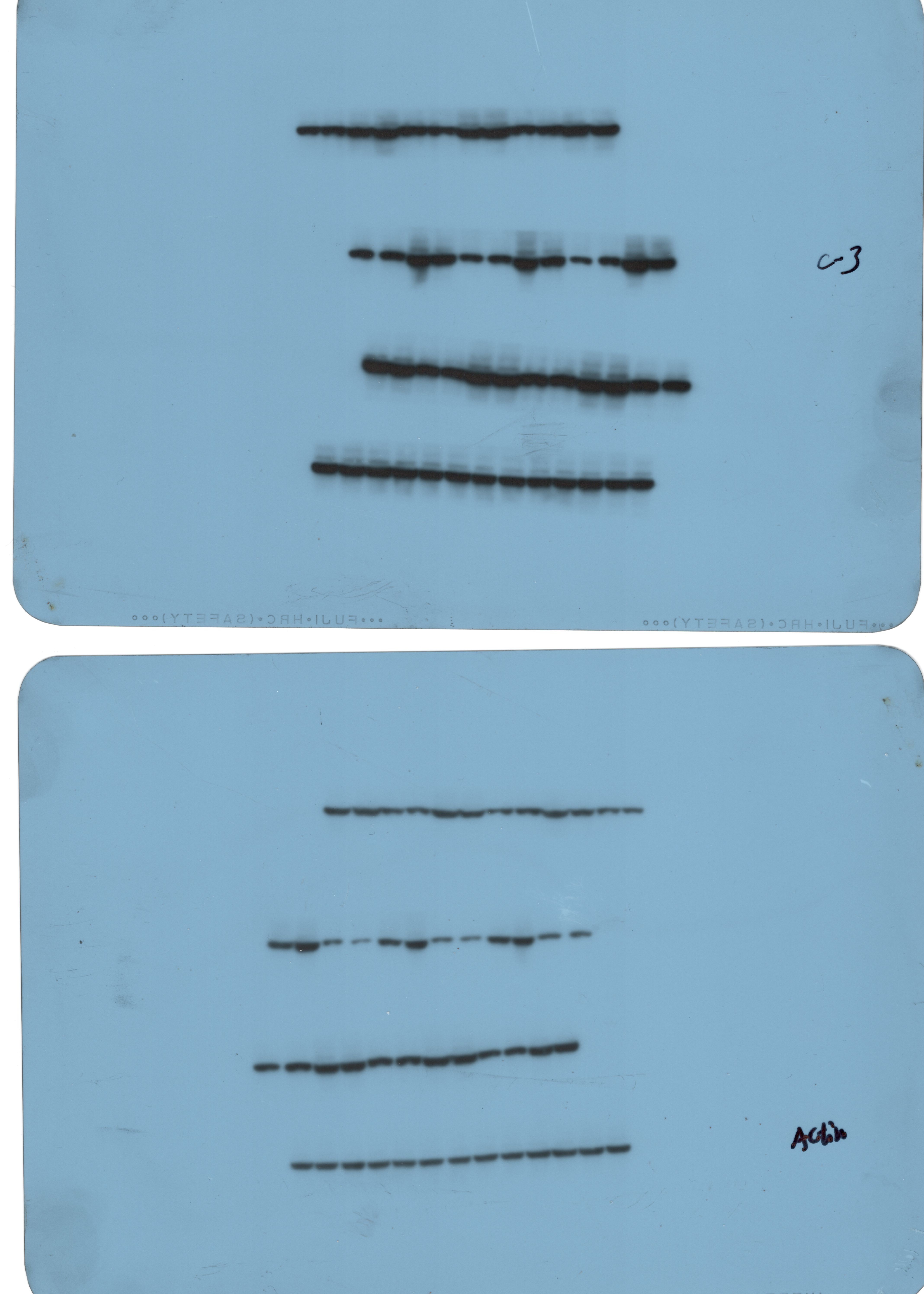

Supplement: S1 File — (ZIP) [file pone.0338802.s006.zip › WB Raw data/Fig S3-2.jpg]

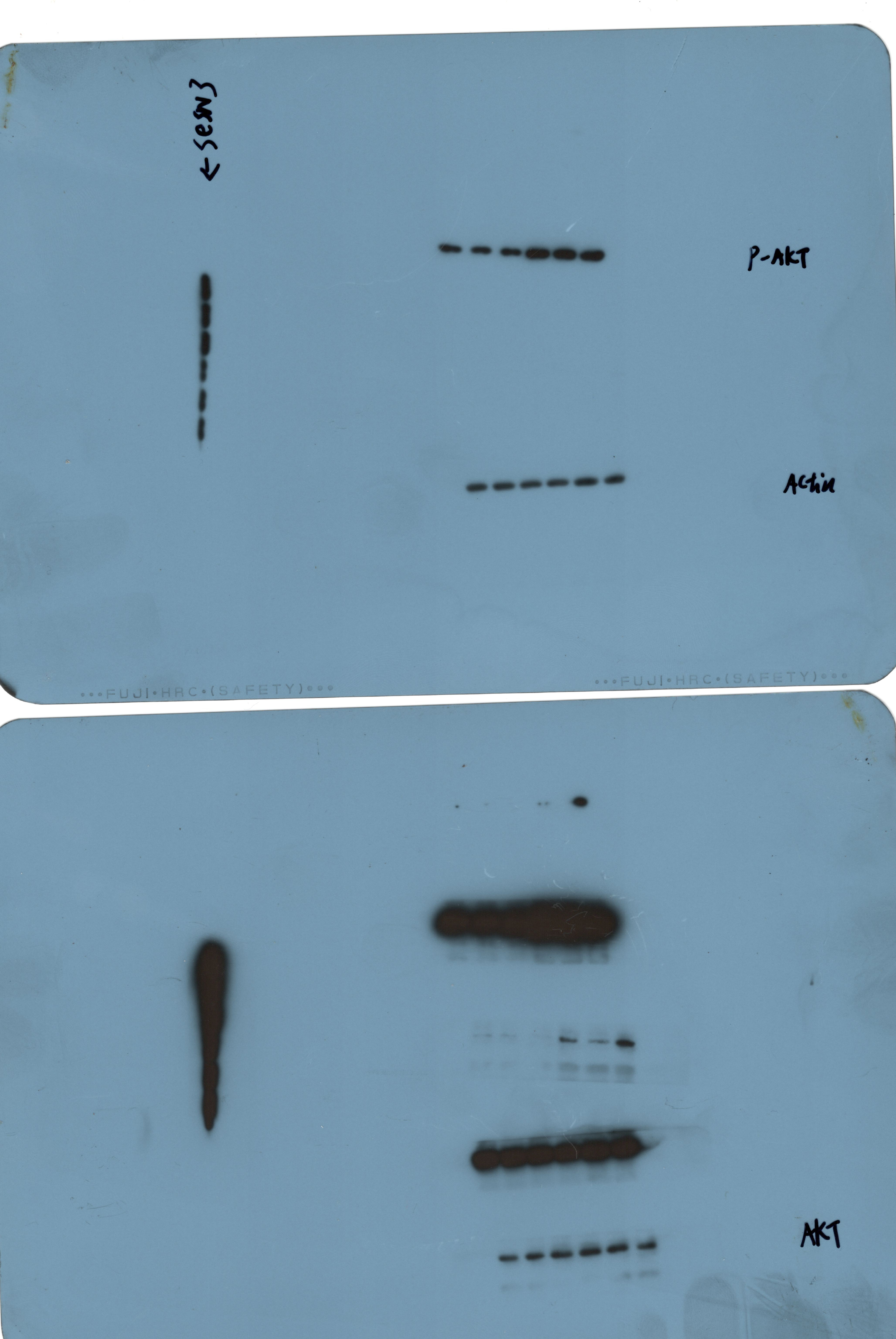

Supplement: S1 File — (ZIP) [file pone.0338802.s006.zip › WB Raw data/Fig S4-1.jpg]

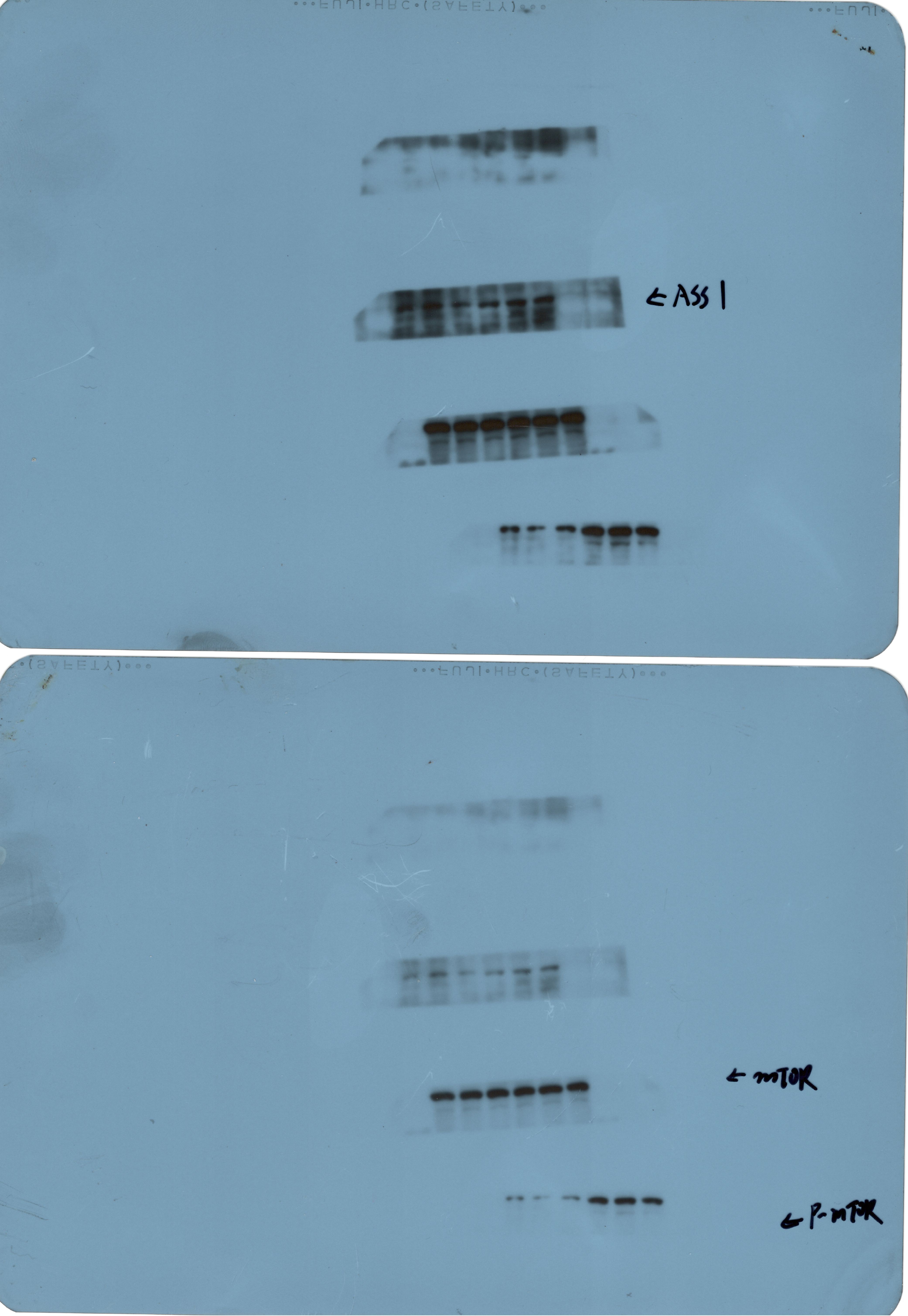

Supplement: S1 File — (ZIP) [file pone.0338802.s006.zip › WB Raw data/Fig S4-2.jpg]

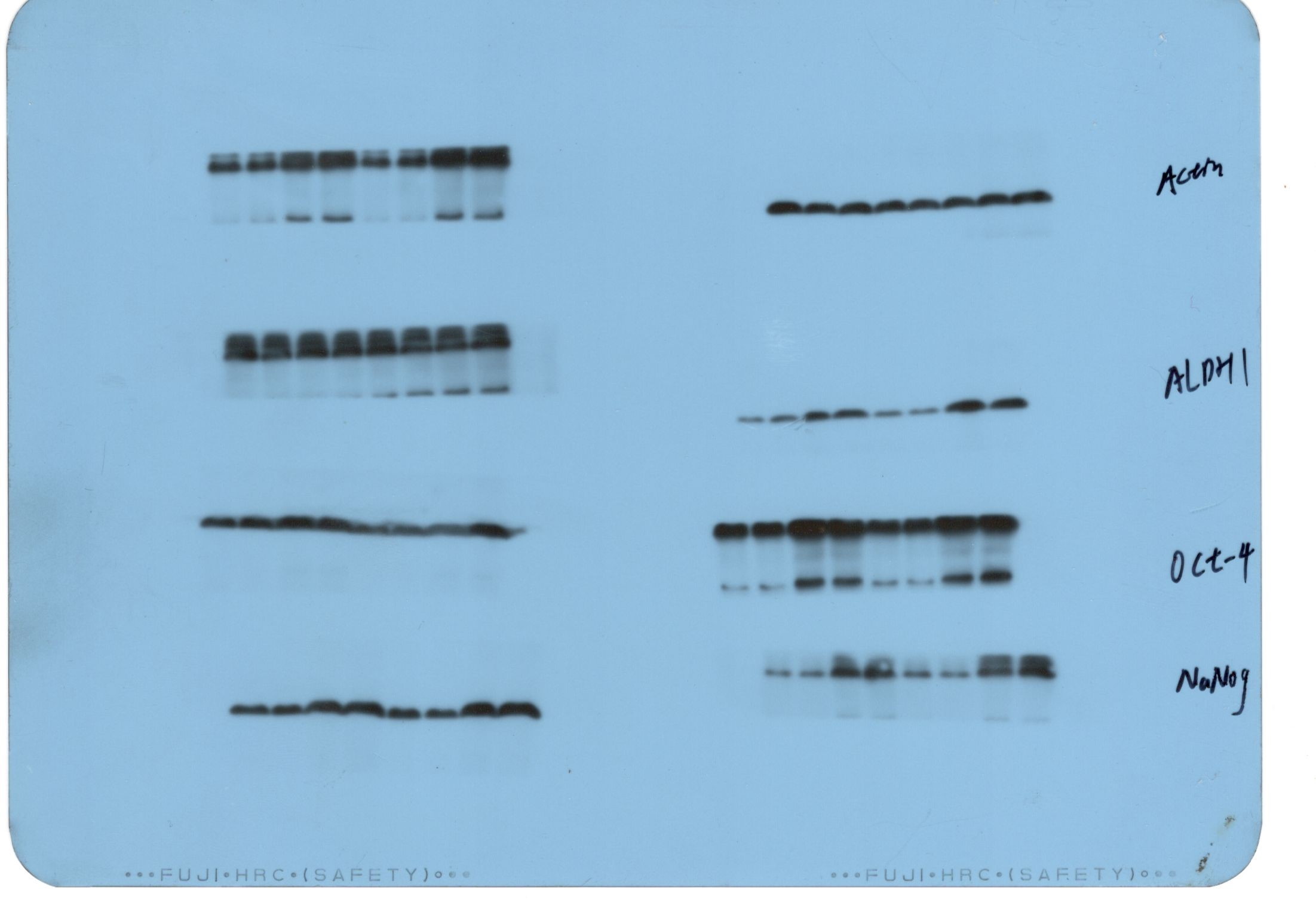

Supplement: S1 File — (ZIP) [file pone.0338802.s006.zip › WB Raw data/Fig2.jpg]

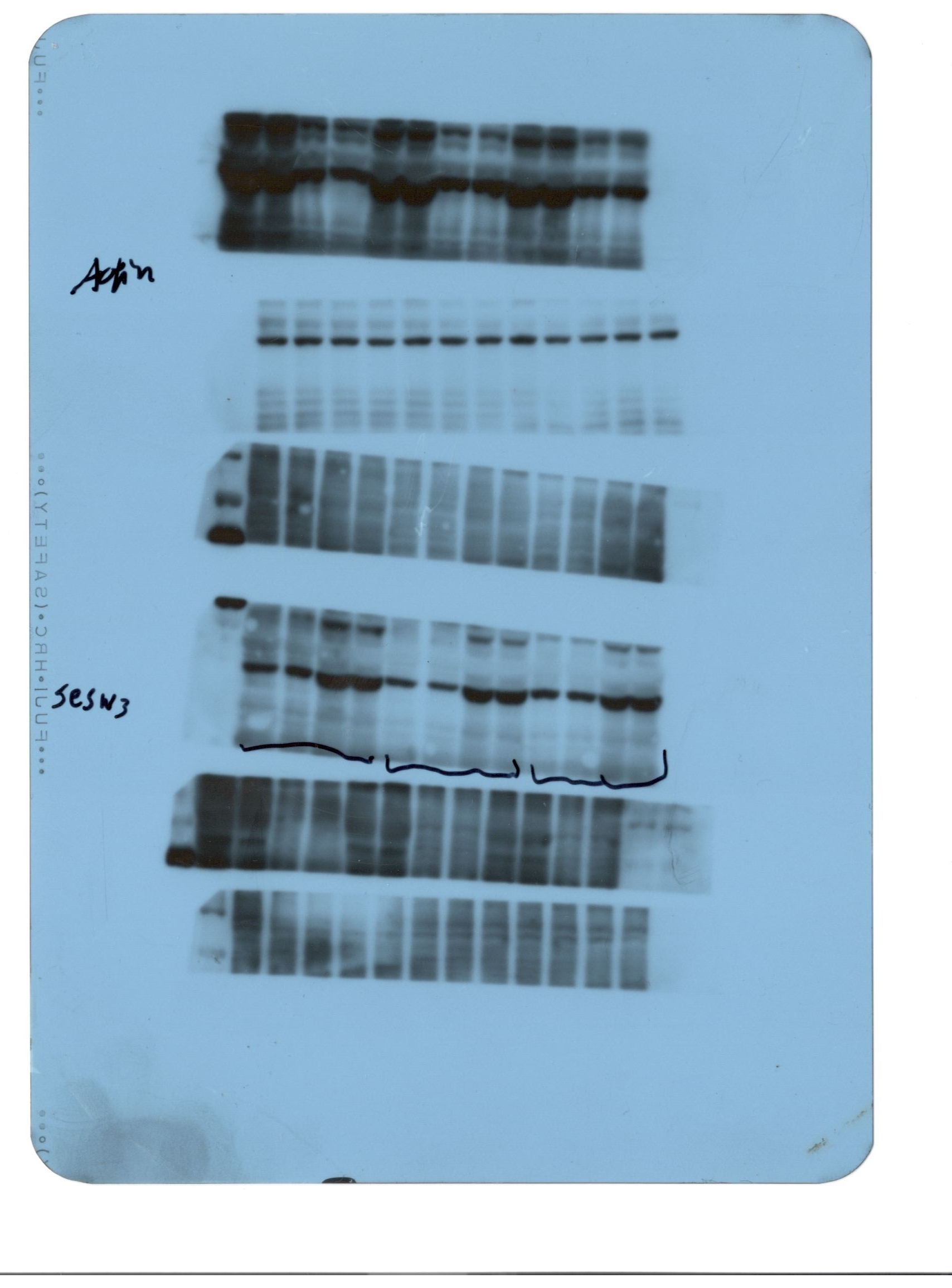

Supplement: S1 File — (ZIP) [file pone.0338802.s006.zip › WB Raw data/Fig3-b.jpg]

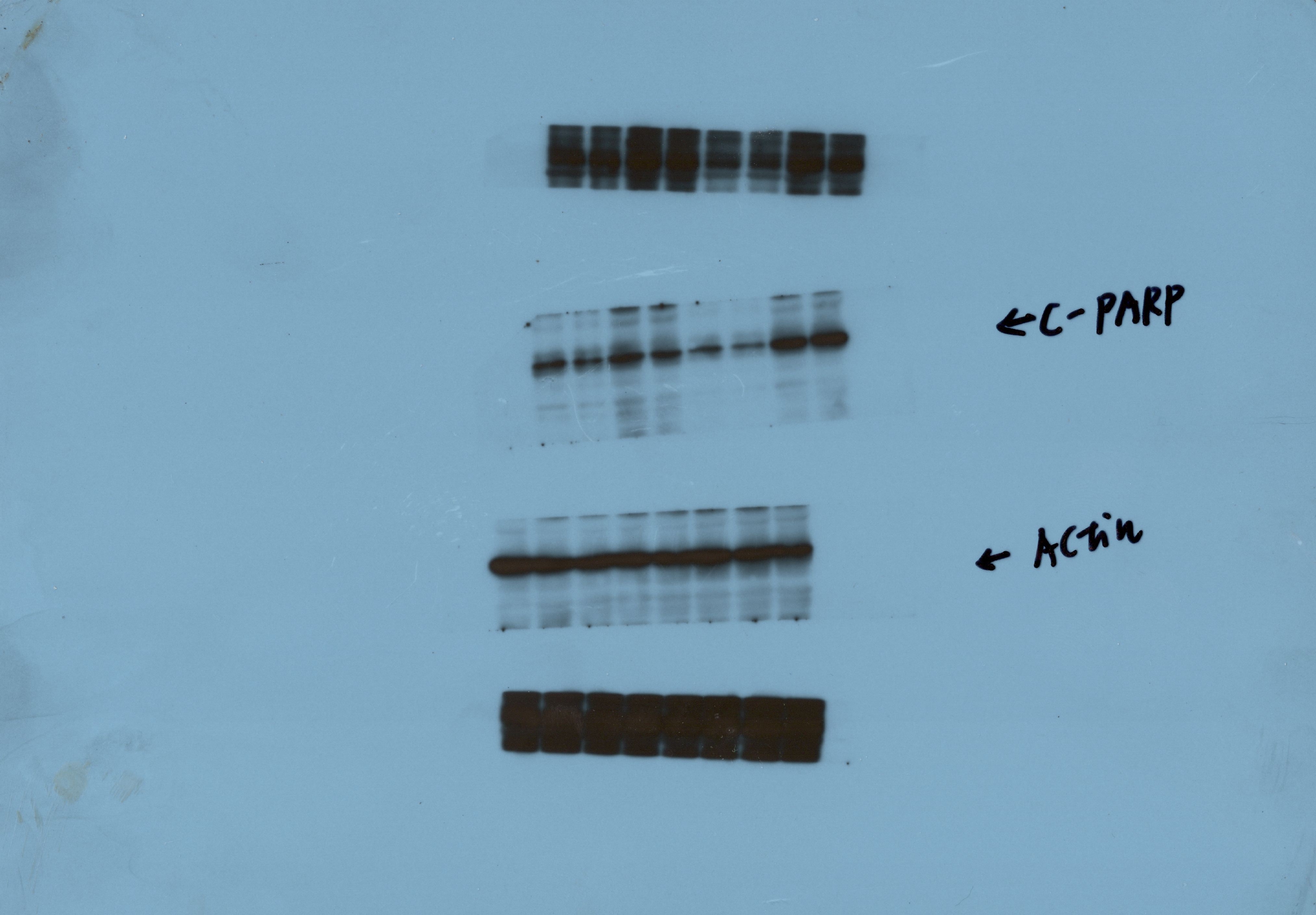

Supplement: S1 File — (ZIP) [file pone.0338802.s006.zip › WB Raw data/Fig3-g.jpg]

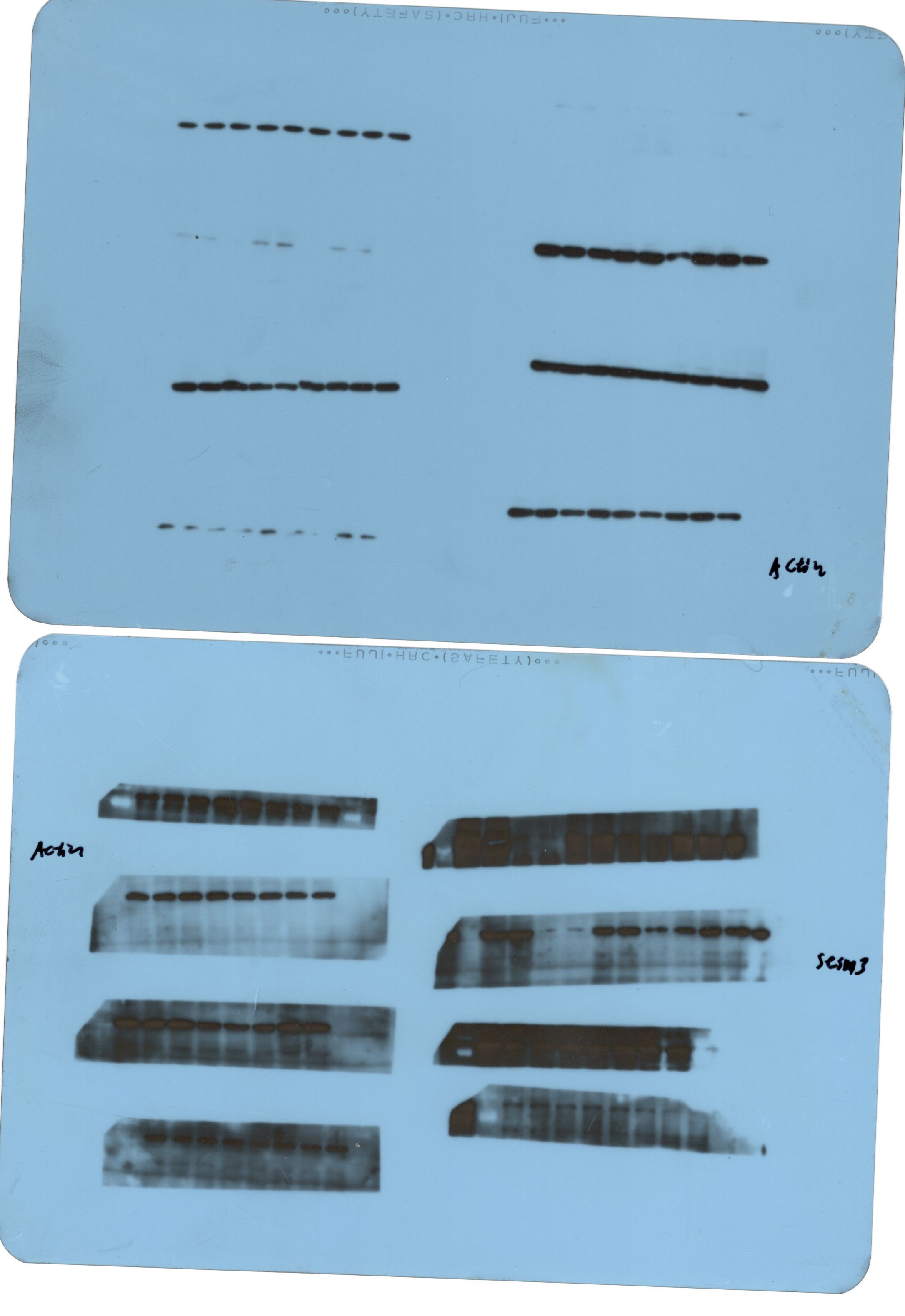

Supplement: S1 File — (ZIP) [file pone.0338802.s006.zip › WB Raw data/Fig4-d.jpg]

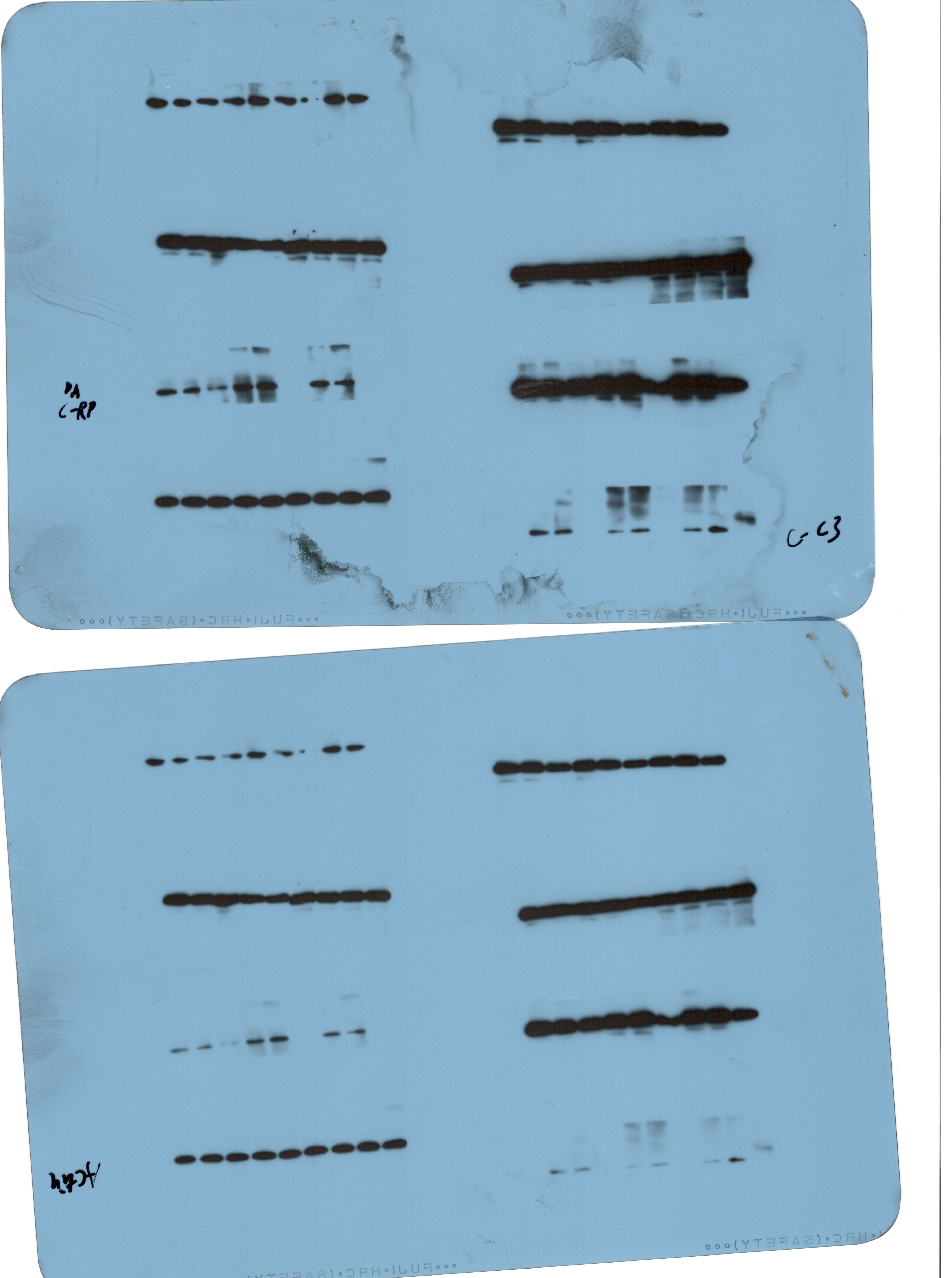

Supplement: S1 File — (ZIP) [file pone.0338802.s006.zip › WB Raw data/Fig5-g.jpg]

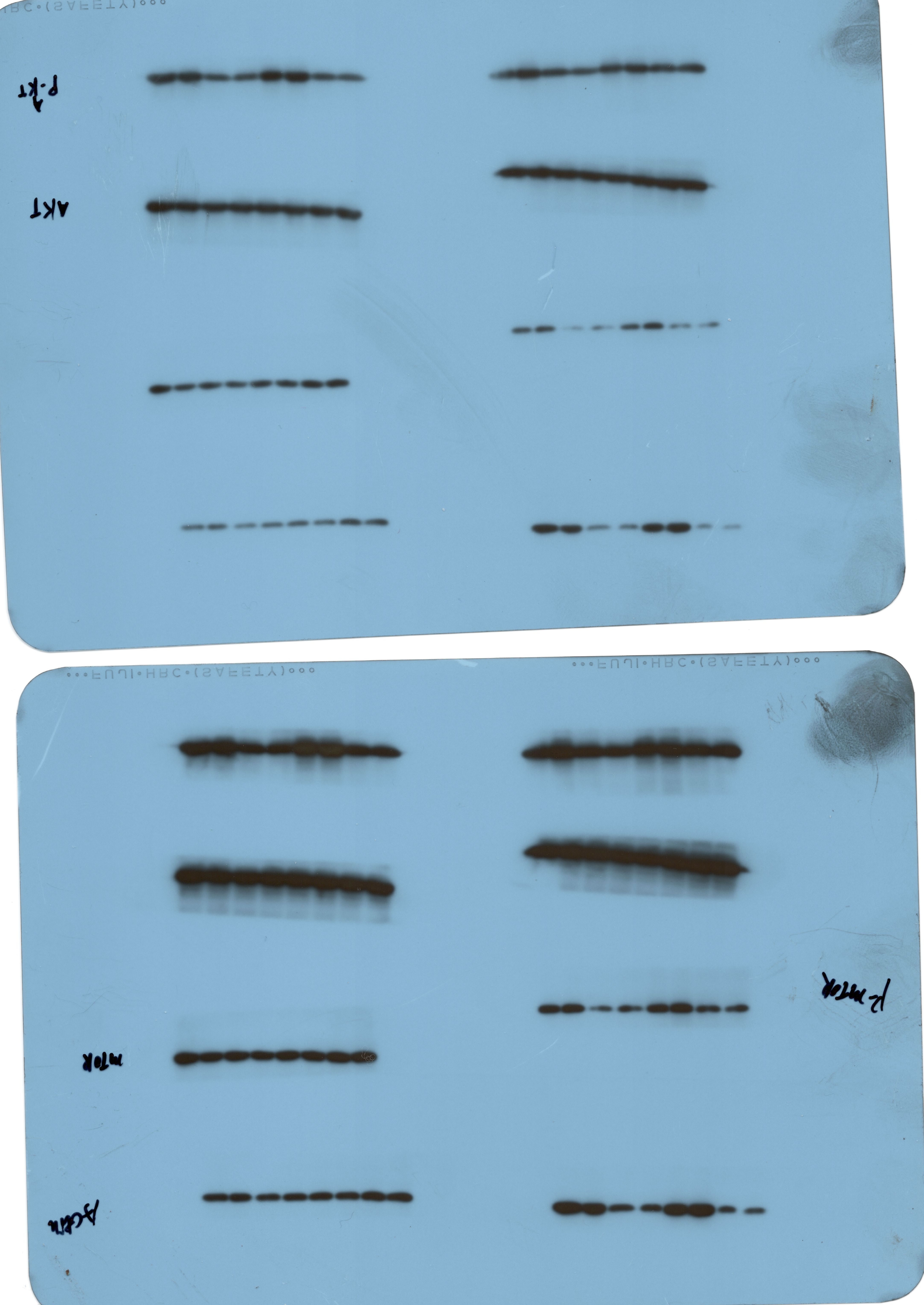

Supplement: S1 File — (ZIP) [file pone.0338802.s006.zip › WB Raw data/Fig6-a.jpg]

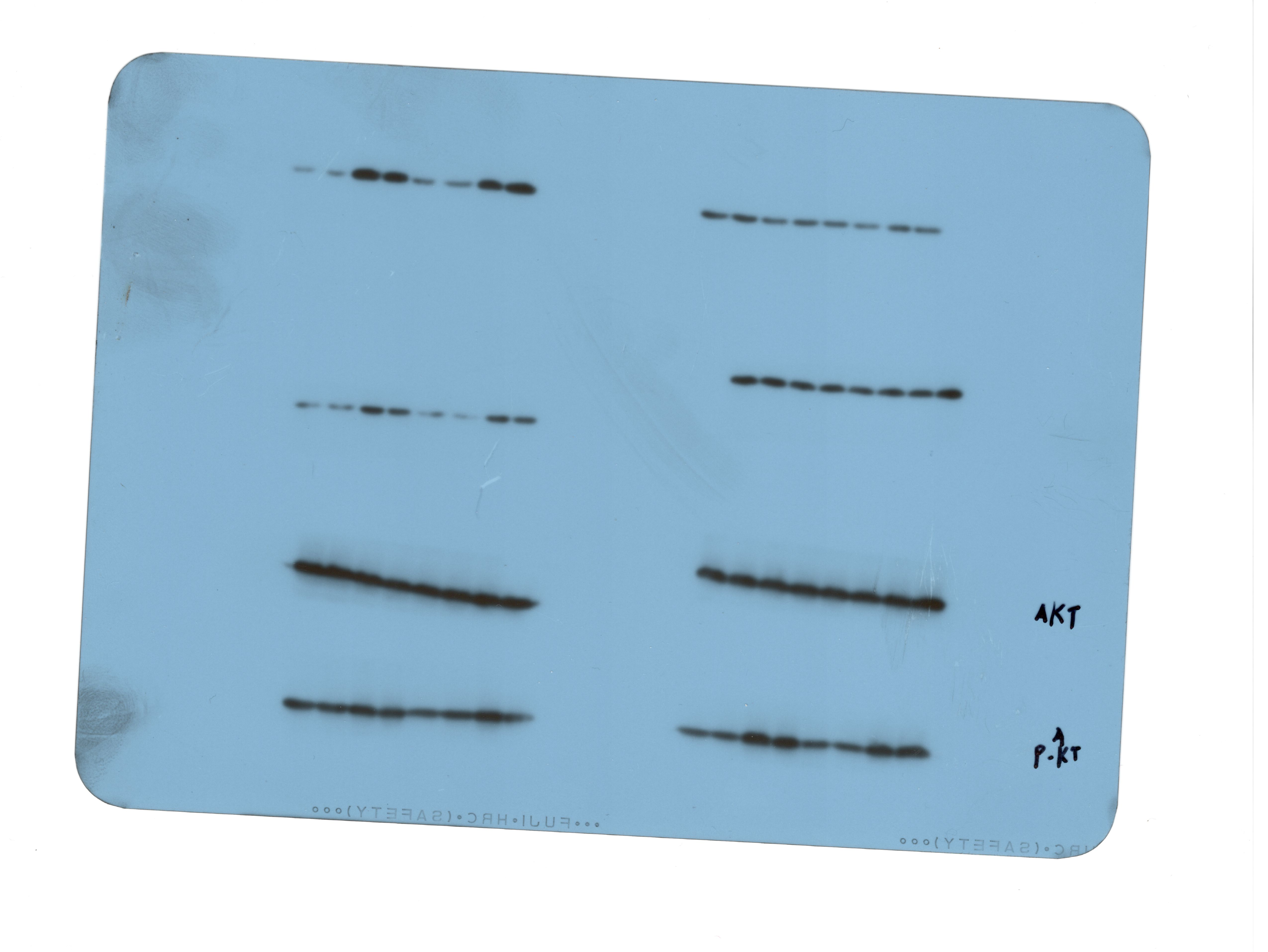

Supplement: S1 File — (ZIP) [file pone.0338802.s006.zip › WB Raw data/Fig6-a1.png]

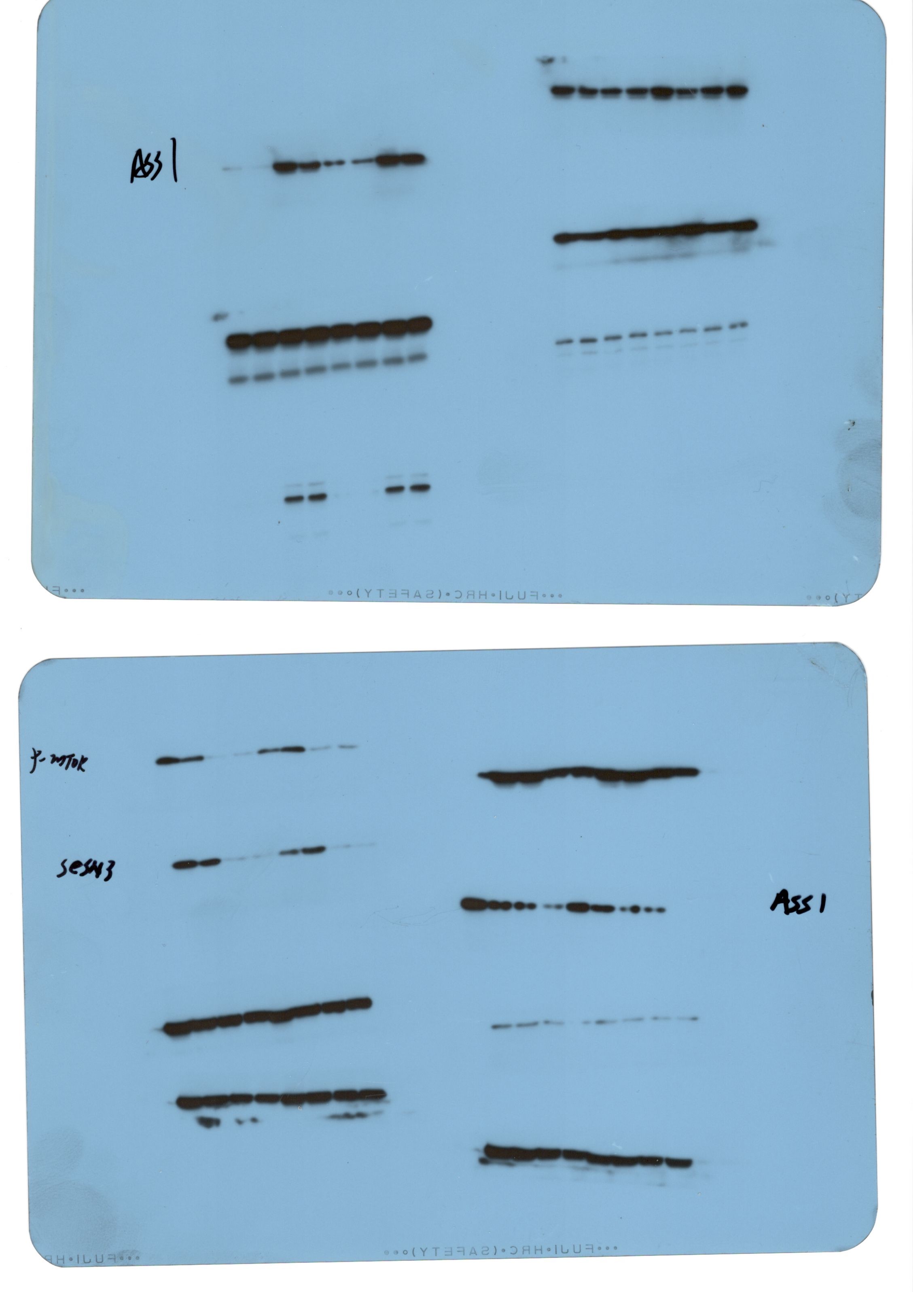

Supplement: S1 File — (ZIP) [file pone.0338802.s006.zip › WB Raw data/Fig6-c&e.jpg]

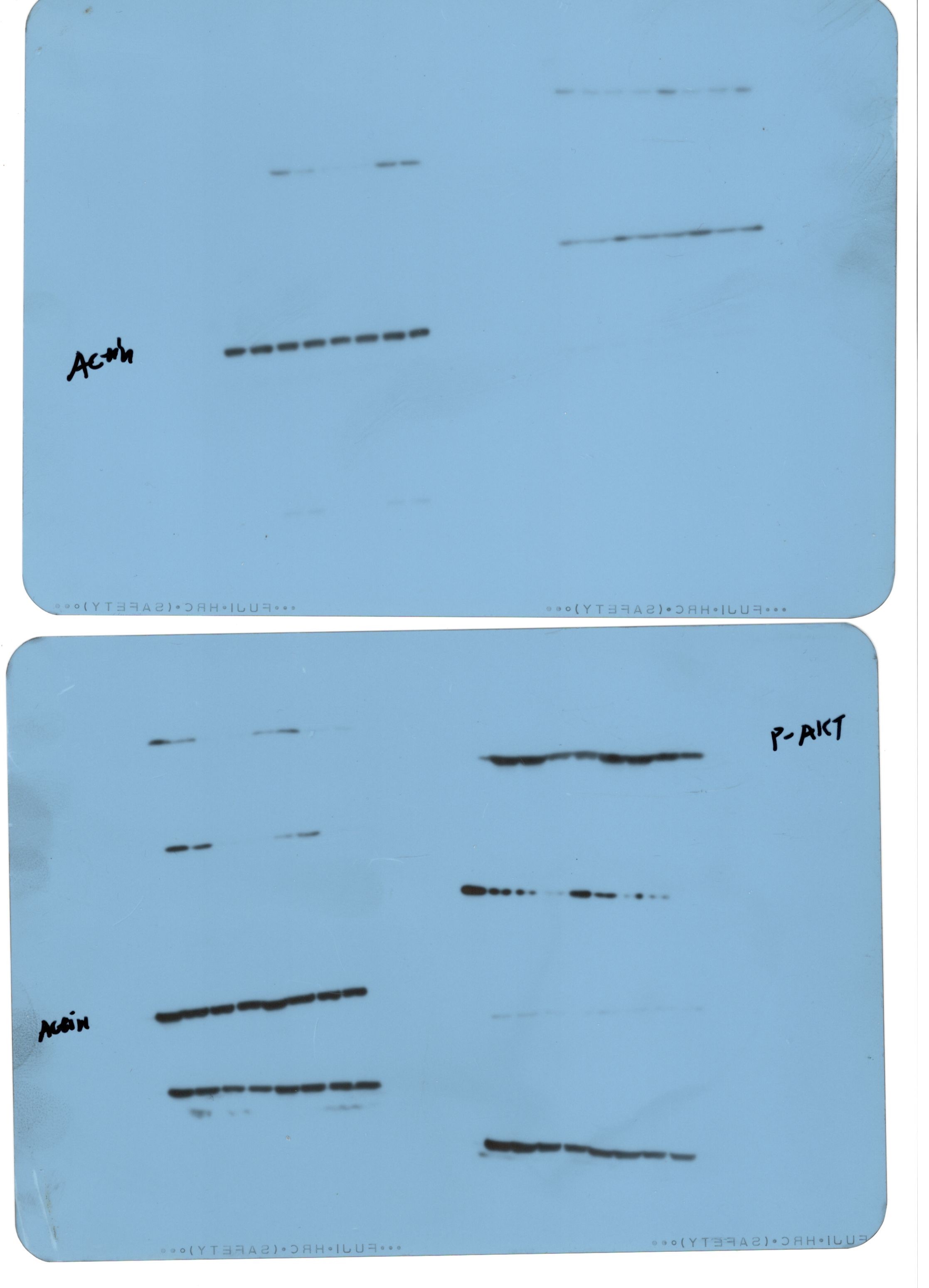

Supplement: S1 File — (ZIP) [file pone.0338802.s006.zip › WB Raw data/Fig6-c&e1.jpg]

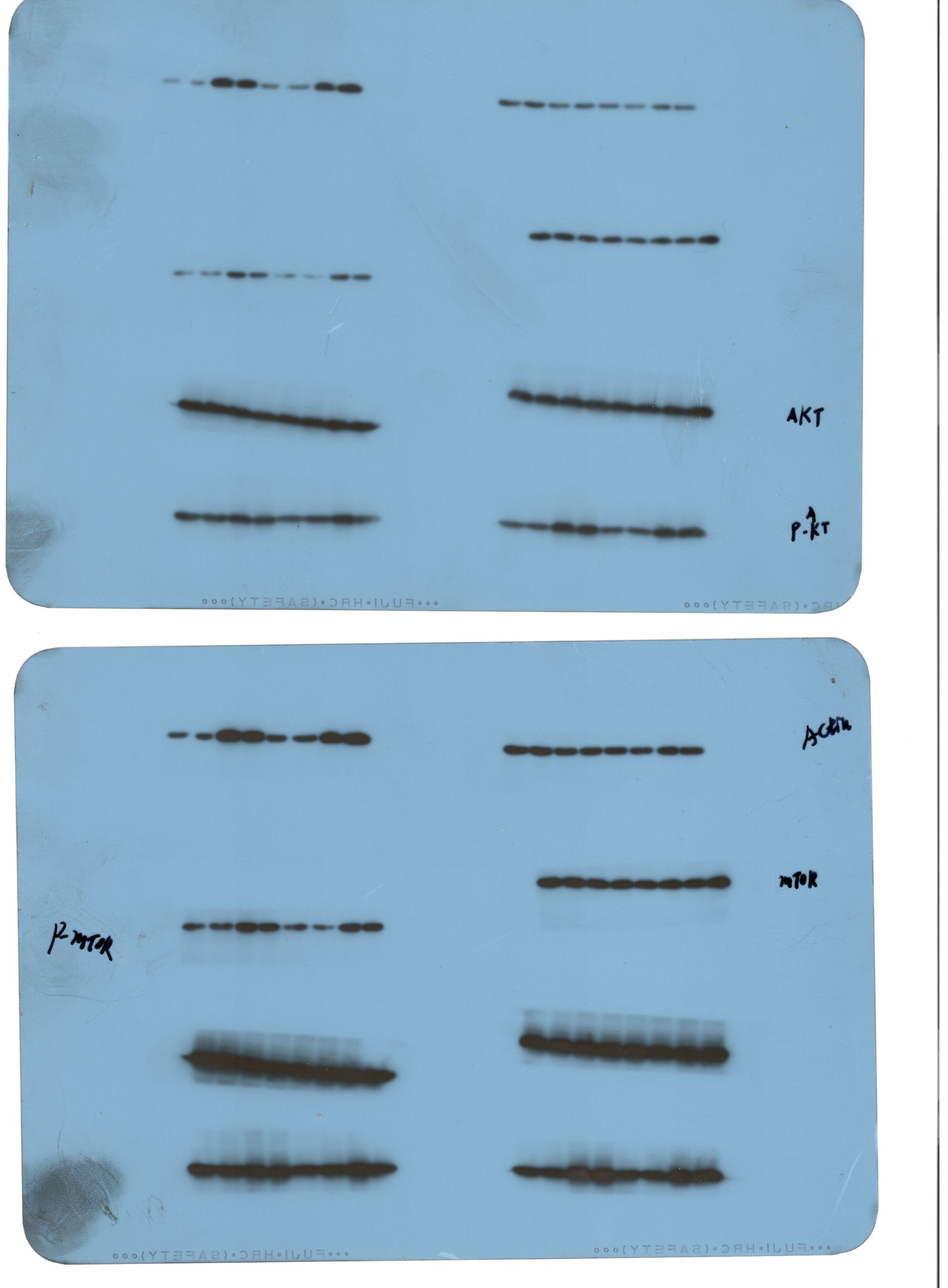

Supplement: S1 File — (ZIP) [file pone.0338802.s006.zip › WB Raw data/Fig6-c&e2.jpg]
