# Supplementary material for: Sestrin3 confers resistance to recombinant human arginase in small cell lung cancer by activating Akt/mTOR/ASS1 axis
Source: PLoS One. 2025 Dec 29;20(12):e0338802. doi: 10.1371/journal.pone.0338802 (PMC12747410; doi:10.1371/journal.pone.0338802)
Supplement: Attachment — Submitted filename: reviewer response PLOS one.pdf [file pone.0338802.s007.pdf]

In this current study "*Sestrin3 confers resistance to recombinant human arginase in small cell lung cancer by activating Akt/mTOR/ASS1 axis*", the authors developed a SCLC cell line resistant to BCT-100 and determined that SESN3 contributes to the resistance phenotype. SCLC is known for acquiring resistance to chemotherapy agents and understanding the mechanism of resistance is vital to identify novel treatment options. The current study requires additional work to differentiate from a previous study by this group which determined that CNTN-1 modulates BCT-100 resistance. The study can be strengthened by addressing the following points:

Major points:

-In a previous publication by your group, H446 and H526 were made resistant to BCT-100 and CNTN-1 was identified as a key gene for resistance. With this current study identifying SESN3, is there a possible connection between SESN3 and CNTN-1 in BCT-100 resistance? Could a double knockout of both genes have an additive effect in sensitizing the resistant cells?

-It is not clear if the expression array was performed with the H446-BR of this study or if the array from the previous publication was datamined to determine SESN3 as a potential candidate for drug resistance. If so, then the expression array data should be displayed as a supplemental table to better justify the rational for exploring SESN3.

-In figure 6 C and E, the western blot for p-AKT and p-mTOR is missing the protein expression of total AKT and mTOR. The p-AKT and P-mTOR should be normalized to the total AKT/mTOR to measure phosphorylation activity.

Minor points

-The standard treatment for SCLC is a combination treatment of etoposide and cisplatin. The H446-BR cell line was shown to be resistant to both etoposide and cisplatin but not in combination treatment of the two drugs. To better mimic the clinical treatment of SCLC, the authors should explore whether H446-BR is resistant to the combination treatment of etoposide and cisplatin.

-In figure 3, wound healing assays were performed with knockdown of SESN3. Did the authors examine a reversal in the expression of the stem cell biomarkers (ALDH1, Oct-4, and Nanog) with shSESN3 though western blot? Likewise, how are EMT markers such as E-cad and N- cad expressed in the resistant cells and does SESN3 affect the expression of these markers?

- Recent studies have identified that SCLC is divided into four main subtypes, ASCL1 (SCLC-A), NEUROD1 (SCLC-N), YAP1 (SCLC-Y) and POU2F3 (SCLC-P). As mentioned as a limitation in the discussion, the current study was performed in one cell line of SCLC. Can the authors provide some additional information regarding the subtype of H446-BR and any insight regarding SESN3 expression across the SCLC subtypes?
