# Supplementary material for: Sestrin3 confers resistance to recombinant human arginase in small cell lung cancer by activating Akt/mTOR/ASS1 axis
Source: PLoS One. 2025 Dec 29;20(12):e0338802. doi: 10.1371/journal.pone.0338802 (PMC12747410; doi:10.1371/journal.pone.0338802)
Supplement: Attachment — Submitted filename: Response to Reviewers.docx [file pone.0338802.s008.docx]

Dear Dr. Alexis G. Murillo Carrasco,

Academic Editor
PLOS ONE,

Thank you very much for reviewing our manuscript. We received useful comments from the reviewers and would like to address them as in the following.

Manuscript title: Sestrin3 confers resistance to recombinant human arginase in small cell lung cancer by activating Akt/mTOR/ASS1 axis.

Reviewer(s)' Comments to Author:

Reviewer: 1

Major Comment:

It is not clear why the cell line H446 was chosen. Are these cells lack the endogenous enzymes argininosuccinate synthetase (ASS) and ornithine transcarbamylase (OTC). Or these cells are dependent on exogenous Arginine?. Thus, it is not clear if it is a cell type dependent study or can be implemented widely to arginase resistance.

Reply: Thanks for your comments. In our previous study (PMID: 31427725), both H446 and H526 cell line were chosen to establish resistant cell lines. However, the tumorigenicity of H526-BR is weak. To determine the phenotype of resistant cells in vivo, we selected H446 cell line in this study.

The expressions of ASS and OTC were rather low in H446 and H526 cells, and this could be referred in our previous study (PMID: 30155941).

Control like H446-BR cells with shNEG and shSESN3 without BCT-100 are missing throughout the experiments. So the reportd conclusions, can be misleading.

Reply: This study aims to explore the different phenotypes of resistant cells and parental cells, and we stated the methods used in figure legend. For example, ROS levels determined by flow cytometry in H446-BR cells infected with shNEG, shSESN3-1, or shSESN3-2, following with incubation with BCT-100 (20 mU/ml, 3 days).

To overrule the influence of increased proliferation on increased migration observed in H446-BR if the cell cycle can be restricted before doing migration assay.

Reply: Thanks for your useful comments. Cell proliferation is correlated with cell cycle, and recombinant human arginase (BCT-100) could induce G1 cell cycle arrest in H446 cell line, which has been displayed in our previous study (PMID: 30155941). We will consider to perform such assay in our future study.

Minor Comment:

The phrase "stemness related genes", rather than stemness should be used.

Reply: We revised it accordingly. Thanks for your advice.

Label must be given for all lanes of western blot experiments in every figure.

Reply: Labels in Western blot were obtained from at least three independent experiments. For consistent layout, we display the representative figures, and all raw data could be referred in supporting information.

Tumor volume in Figure 4B should display results of all 4 conditions.

Reply: The main aim of Figure 4 is to compare the different cell growth rate in vivo, so we only selected shNEG and sh-SESN3-1 in Figure 4B.

All the figures should be of high resolution. Flow cytometry data and cell percentage are barely visible.

Reply: The original figures with high resolution could be obtained in supporting information.

Reviewer: 2

Major comments

1. Validate SESN3 expression and ASS1/p-Akt/p-mTOR levels in at least one additional SCLC line (ideally another arginase-resistant model) to show generalisability.

Reply: Thanks for your comments. Actually, we have another arginase-resistant cell line (H526-BR), we did not display the characteristic of H526-BR due to its weak tumorigenicity. Currently, we conduct additional experiment to demonstrate the expressions of ASS1/p-Akt/p-mTOR in Figure S4.

2. Provide human evidence: analyse publicly available SCLC datasets (e.g., CCLE, TCGA) or patient biopsies for SESN3 and ASS1 expression and correlate with reported arginase sensitivity where possible.

Reply: Currently, limited patients with arginase resistance are available for further study. And we discussed it and acknowledged this as relevant limitation in our study.

3. Clarify SESN3’s position in the pathway: does SESN3 interact physically or transcriptionally with Akt or ASS1? Rescue experiments using constitutively active Akt or ectopic ASS1 after SESN3 knock-down would strengthen causality.

Reply: In supplementary figure 3A, overexpression of SESN3 in H446 could induce ASS1, which was line with H446-BR. In figure 6C, silencing SESN3 could down-regulate the levels of ASS1, p-Akt, and p-mTOR. Therefore, we believe SESN3 was located the upstream of this cell signaling pathway. However, RNA pull-down and Co-IP were needed to further confirm our view. We rephase our conclusion as” our results demonstrated that high expression of SESN3 is associated with the resistance of SCLC cells to recombinant human arginase”.

4. Improve statistical reporting: specify exact n for each experiment, define error bars, state tests used, verify that multiple-comparison corrections were applied where appropriate.

Reply: Thanks for your comment. We stated statistical reporting on figure legends.

In figure 1, 2, 3, 5, and 6, “Error bars indicate the mean (SD); n=3 or 4. P-values were determined using one-way ANOVA (**P*<0.05, ***P*<0.01, ****P*<0.001).” was verified.

In figure 4, “Error bars indicate the mean (SEM); *n*=8. P-values were determined using a Mann-Whitney *U* test” was verified.

Minor comments

1. Provide shRNA target sequences (unless proprietary restrictions are documented) to ensure reproducibility.

Reply: The SESN3 shRNA (h) Lentiviral Particles were purchased from Santa Cruz Biotechnology, and the sequences we used were as followed:

SESN3-1：

Forward oligo: 5’ CCGG—GCTAGACATCAGTGTTCTTAC—CTCGAG—GTAAGAACACTGATGTCTAGC—TTTTTG 3’

SESN3-2:

Forward oligo: 5’ CCGG—GCGTTTGTGATCTTGCTAATG—CTCGAG—CATTAGCAAGATCACAAACGC—TTTTTG 3’

2. Confirm normality assumptions before applying t-tests; if data are non-parametric, use appropriate tests.

Reply: Thanks for your useful advice. We confirmed it and used a Mann-Whitney *U* test where data are non-parametric.

3. The term “aggressiveness” (Abstract/Results) is vague; replace with specific phenotypes (e.g., migration, proliferation).

Reply: We replaced it with migration in Abstract part. “More aggressiveness” is replaced by “enhanced migration ability”.

4. Correct typographical errors (e.g., “in intro” should read “in vitro”; “Moclecular weight” in Table 1).

Reply: Thank you. And we revised these accordingly.

5. Discussion: moderate the claim that SESN3 “induces” Akt/mTOR activation unless direct evidence is provided; consider phrasing as “is associated with”.

Reply: Thanks for your rigorous comment. We revised “induced” to “is associated with” in Discussion part.

Reviewer: 3

Major points:

-In a previous publication by your group, H446 and H526 were made resistant to BCT-100 and CNTN-1 was identified as a key gene for resistance. With this current study identifying SESN3, is there a possible connection between SESN3 and CNTN-1 in BCT-100 resistance? Could a double knockout of both genes have an additive effect in sensitizing the resistant cells?

Reply: Thanks for your valuable advice. We performed a double knockout of SESN3 and CNTN-1 to confirm the synergistic effect in sensitizing the resistant cells. Notably, the wound healing rate in double knockout group (35.0±13.2%) was lower than single knockout group (66.3±3.5% and 70.7±12.4%). And we displayed this finding in Result part (S1 Figure).

-It is not clear if the expression array was performed with the H446-BR of this study or if the array from the previous publication was datamined to determine SESN3 as a potential candidate for drug resistance. If so, then the expression array data should be displayed as a supplemental table to better justify the rational for exploring SESN3.

Reply: Thanks for your comment. The expression of SESN3 in H446-BR cell line was displayed in Figure 3, which displayed high level in resistant cells. Besides, SESN3 was also associated with several cancer cells resistance to chemotherapeutic drugs (PMID: 25973791, PMID: 39863613, PMID: 29152111).

-In figure 6 C and E, the western blot for p-AKT and p-mTOR is missing the protein

expression of total AKT and mTOR. The p-AKT and P-mTOR should be normalized to the total AKT/mTOR to measure phosphorylation activity.

Reply: Thanks for your advice. We conducted additional experiment to test the expressions of total AKT and mTOR and revised Figure 6 accordingly.

Minor points

-The standard treatment for SCLC is a combination treatment of etoposide and cisplatin. The H446-BR cell line was shown to be resistant to both etoposide and cisplatin but not in combination treatment of the two drugs. To better mimic the clinical treatment of SCLC, the authors should explore whether H446-BR is resistant to the combination treatment of etoposide and cisplatin.

Reply: Thanks for your useful comment. We admit this is a study design flaw, and we will use combination treatment of etoposide and cisplatin to test the drug resistance of H446-BR cell line in the future study.

-In figure 3, wound healing assays were performed with knockdown of SESN3. Did the authors examine a reversal in the expression of the stem cell biomarkers (ALDH1, Oct-4, and Nanog) with shSESN3 though western blot? Likewise, how are EMT markers such as E-cad and N- cad expressed in the resistant cells and does SESN3 affect the expression of these markers?

Reply: Thanks for your comment. We conducted additional assay to test the levels of ALDH1, Oct-4, Nanog, and N-cadherin in shNEG and shSESN3 groups, which meet the hypotheses. And we revised Fig 3 accordingly.

- Recent studies have identified that SCLC is divided into four main subtypes, ASCL1

(SCLC-A), NEUROD1 (SCLC-N), YAP1 (SCLC-Y) and POU2F3 (SCLC-P). As mentioned as a limitation in the discussion, the current study was performed in one cell line of SCLC. Can the authors provide some additional information regarding the subtype of H446-BR and any insight regarding SESN3 expression across the SCLC subtypes?

Reply: Thanks for your useful comment. We employed another resistant SCLC cell line (H526-BR) to reconfirm our finding with high expressions of expressions of SESN3 and ASS1/p-Akt/p-mTOR axis as displayed in S1 Figure.

Journal Requirements:

1. Please ensure that your manuscript meets PLOS ONE's style requirements, including those for file naming. The PLOS ONE style templates can be found at https://journals.plos.org/plosone/s/file?id=wjVg/PLOSOne_formatting_sample_main_body.pdf and https://journals.plos.org/plosone/s/file?id=ba62/PLOSOne_formatting_sample_title_authors_affiliations.pdf

 Reply: We revised our manuscript accordingly, please inform me if revision needed.

2. PLOS ONE now requires that authors provide the original uncropped and unadjusted images underlying all blot or gel results reported in a submission’s figures or Supporting Information files. This policy and the journal’s other requirements for blot/gel reporting and figure preparation are described in detail at https://journals.plos.org/plosone/s/figures#loc-blot-and-gel-reporting-requirements and https://journals.plos.org/plosone/s/figures#loc-preparing-figures-from-image-files. When you submit your revised manuscript, please ensure that your figures adhere fully to these guidelines and provide the original underlying images for all blot or gel data reported in your submission. See the following link for instructions on providing the original image data: https://journals.plos.org/plosone/s/figures#loc-original-images-for-blots-and-gels.
In your cover letter, please note whether your blot/gel image data are in Supporting Information or posted at a public data repository, provide the repository URL if relevant, and provide specific details as to which raw blot/gel images, if any, are not available. Email us at [plosone@plos.org](mailto:plosone@plos.org) if you have any questions.

 Reply:

The original uncropped and unadjusted images included in our study was submitted in system.

3. To comply with PLOS One submissions requirements, in your Methods section, please provide additional information regarding the experiments involving animals and ensure you have included details on methods of anesthesia and/or analgesia, and efforts to alleviate suffering.

 Reply: In our Methods section, we stated “Mice were euthanized using sodium pentobarbital (i.p., 180 mg/kg) when the tumor volume over 800 mm^3^, which was regarded as a humane endpoint. Once animals reached endpoint criteria, the amount of time elapsed before euthanasia was less than 10 min. Cervical dislocation was used to confirm animal death without breathing or heartbeat. Animal health and behavior were monitored daily by trained staff to ensure well-being. No unexpected deaths occurred during the study.”

4. Your ethics statement should only appear in the Methods section of your manuscript. If your ethics statement is written in any section besides the Methods, please delete it from any other section.

 Reply: We revised it accordingly.

5. We note that your Data Availability Statement is currently as follows: All relevant data are within the manuscript and its Supporting Information files.

Please confirm at this time whether or not your submission contains all raw data required to replicate the results of your study. Authors must share the “minimal data set” for their submission. PLOS defines the minimal data set to consist of the data required to replicate all study findings reported in the article, as well as related metadata and methods (https://journals.plos.org/plosone/s/data-availability#loc-minimal-data-set-definition).

For example, authors should submit the following data:

- The values behind the means, standard deviations and other measures reported;
- The values used to build graphs;
- The points extracted from images for analysis.

Authors do not need to submit their entire data set if only a portion of the data was used in the reported study.

If your submission does not contain these data, please either upload them as Supporting Information files or deposit them to a stable, public repository and provide us with the relevant URLs, DOIs, or accession numbers. For a list of recommended repositories, please see https://journals.plos.org/plosone/s/recommended-repositories.

If there are ethical or legal restrictions on sharing a de-identified data set, please explain them in detail (e.g., data contain potentially sensitive information, data are owned by a third-party organization, etc.) and who has imposed them (e.g., an ethics committee). Please also provide contact information for a data access committee, ethics committee, or other institutional body to which data requests may be sent. If data are owned by a third party, please indicate how others may request data access.

 Reply: We submitted all raw data in submission system.

6. Thank you for stating the following financial disclosure: Guangdong Basic and Applied Basic Research Foundation(Grant No. 2024A1515012821). Please state what role the funders took in the study. If the funders had no role, please state: "The funders had no role in study design, data collection and analysis, decision to publish, or preparation of the manuscript."
If this statement is not correct you must amend it as needed.
Please include this amended Role of Funder statement in your cover letter; we will change the online submission form on your behalf.

 Reply: We stated “The funder (Guangdong Basic and Applied Basic Research Foundation, Grant No. 2024A1515012821) had no role in study design, data collection and analysis, decision to publish, or preparation of the manuscript” in Cover Letter.

7. Please update your submission to use the PLOS LaTeX template. The template and more information on our requirements for LaTeX submissions can be found at http://journals.plos.org/plosone/s/latex.

 Reply: We tried to use LaTeX submission, and please note me if revision needed.

8. Please amend your list of authors on the manuscript to ensure that each author is linked to an affiliation. Authors’ affiliations should reflect the institution where the work was done (if authors moved subsequently, you can also list the new affiliation stating “current affiliation:….” as necessary).

 Reply: We revised it accordingly.

9. We notice that your supplementary figures are uploaded with the file type 'Figure'. Please amend the file type to 'Supporting Information'. Please ensure that each Supporting Information file has a legend listed in the manuscript after the references list.

 Reply: We revised it accordingly.

10. If the reviewer comments include a recommendation to cite specific previously published works, please review and evaluate these publications to determine whether they are relevant and should be cited. There is no requirement to cite these works unless the editor has indicated otherwise.

 Reply: Not applicable.

Sincerely,

Shi Xu

Please address the correspondence to:

Shi Xu, PhD

Associate researcher

Shenzhen Longhua District Central Hospital,

Shenzhen,

Guangdong,

China

Email: xushi_cn@163.com	Tel: (86) 755 2801 4167
