# Supplementary material for: Sestrin3 confers resistance to recombinant human arginase in small cell lung cancer by activating Akt/mTOR/ASS1 axis
Source: PLoS One. 2025 Dec 29;20(12):e0338802. doi: 10.1371/journal.pone.0338802 (PMC12747410; doi:10.1371/journal.pone.0338802)
Supplement: Attachment — Submitted filename: Response_to_Reviewers_auresp_2.docx [file pone.0338802.s009.docx]

Dear Dr. Alexis G. Murillo Carrasco,

Academic Editor
PLOS ONE,

Thank you very much for reviewing our manuscript. We received useful comments from the reviewers and would like to address them as in the following.

Manuscript title: Sestrin3 confers resistance to recombinant human arginase in small cell lung cancer by activating Akt/mTOR/ASS1 axis.

Reviewer(s)' Comments to Author:

In particular, the main features of the small cell lung cancer (SCLC) cell line used should be clearly characterized. Based on an expression profiling tool for cancer cell lines, such as ShinyThor (https://doi.org/10.1093/bioadv/vbaf061), this cell line appears to be among the highest SESN3-expressing models. However, it originates from a pleural effusion, which represents a metastatic sample, and this should be explicitly stated in the manuscript.

Reply：

Thanks for your very useful comment for our study. We used ShinyThor to confirm the basal level of SESN3 in H446 cell. We discuss this issue in our Discussion Part as consider it as the limitation of our study. “H446 cell line originates from a pleural effusion, which represents a metastatic sample. Based on an expression profiling tool for cancer cell lines, such as ShinyThor, H446 appears to be among the highest SESN3-expressing models [42]. It might indicate SESN3 has distinct inducible ability.”

Additionally, since you reanalyze data from a previous study (ref19) to assess SESN3 levels, the processed dataset should be deposited in a publicly accessible repository (e.g., Gene Expression Omnibus), and the associated accession code must be included in the submission via the data availability statement. To better reflect clinical relevance, it is also recommended to include a patient-level analysis of SESN3 expression, along with other related genes, in relation to patient survival.

Reply：

Thanks for your comment. The raw data of RNA was deposited in Dr Jamse Ho (Professor in HKU). In this study, we used Western blot and qPCR to confirm the high level of SESN3 in H446-BR cells. However, to comply with our policy, we delete the RNA sequencing analysis statement and Ref19 accordingly. Currently, we have very limited patients with BCT-100 resistance to further confirm the relation of SESN3 to patient survival. And we have discussed this issue as regarded it as one of limitations in our manuscript.

Sincerely,

Shi Xu

Please address the correspondence to:

Shi Xu, PhD

Associate researcher

Shenzhen Longhua District Central Hospital,

Shenzhen,

Guangdong,

China

Email: xushi_cn@163.com	Tel: (86) 755 2801 4167
