# Supplementary material for: Sestrin3 confers resistance to recombinant human arginase in small cell lung cancer by activating Akt/mTOR/ASS1 axis
Source: PLoS One. 2025 Dec 29;20(12):e0338802. doi: 10.1371/journal.pone.0338802 (PMC12747410; doi:10.1371/journal.pone.0338802)
Supplement: Attachment — Submitted filename: Response_to_Reviewers_auresp_3.docx [file pone.0338802.s010.docx]

Dear Dr. Alexis G. Murillo Carrasco,

Academic Editor
PLOS ONE,

Thank you very much for reviewing our manuscript. We received useful comments from the reviewers and would like to address them as in the following.

Manuscript title: Sestrin3 confers resistance to recombinant human arginase in small cell lung cancer by activating Akt/mTOR/ASS1 axis.

Reviewer(s)' Comments to Author:

Reviewer #1: Authors have made sufficient corections and performed new experiments. It is adviced to please extensively proofread the manuscript and correct the phrasing of sentences.

Reply: Thanks for your kindly review.

Reviewer #2: (1) Provide a minimal but concrete public-data analysis demonstrating the prevalence and clinical context of SESN3 and ASS1 expression in SCLC—e.g., quantify transcript levels across CCLE SCLC lines and at least one patient cohort (GEO/ArrayExpress), report effect sizes and FDR-adjusted P values, and include a brief survival or subtype association if available.

Reply: Thanks for your valuable advice. We made gene correlation analysis between SESN3 and ASS1 by GePIA cancer database (Figure 1). It seems that these two genes have not significant relevance within normal lung cancer. Besides, the SESN3 and ASS1 expressions in SCLC cell lines vary significantly (Figure 2). However, these data were analyzed in normal SCLC, which may differ from BCT-100 resistant context. Due to the limited clinical samples, currently we cannot explore the relation of genes level and brief survival, which is one of limitations in this study.

(2) Establish pathway order with a mechanistic rescue: in SESN3-knockdown H446-BR (and/or H526-BR), restore signaling using constitutively active Akt or ectopic ASS1, then show recovery of mTORC1 readouts (p-S6K, p-4EBP1), ASS1 protein, and functional resistance to BCT-100.

Reply: Thanks for your suggestion. In our current study, we already found SESN3-knockdown could reserve phenotype of drug resistance in H446-BR cells. To confirm our findings, we used Akt activator SC79 in H446-BR cells transfected with shSESN3, with recovery of p-Akt, p-mTOR, and ASS1 (S4 Fig).

Reviewer #3: Dear authors,

Thank you for the response to my comments. My comments on the manuscript have been addressed, and the manuscript is ready to be published. I appreciate the additional response in addressing the limitations of the study. One minor comment regards the following sentence under the section of “SESN3 silencing inhibited the cellular migration and promoted cellular apoptosis”, where the underlined words appear to be in a different font than the rest of the manuscript: “. In line with previous results, ALDH1, Oct-4, Nanog, and N-cadherin were suppressed in H446-BR cell line infected with SESN3 (Fig 3F).”

Reply: Thanks for your kindly review. We revised the font accordingly.

Sincerely,

Shi Xu

Please address the correspondence to:

Shi Xu, PhD

Associate researcher

Shenzhen Longhua District Central Hospital,

Shenzhen,

Guangdong,

China

Email: xushi_cn@163.com	Tel: (86) 755 2801 4167

Figures to address reviewer’s comments.

Figure 1 Gene correlation analysis between SESN3 and ASS1.

Gene Expressions

|  | ASS1 | SESN3 |
| --- | --- | --- |
| NCIH1694 | 15.677 | 4.33988 |
| NCIH1184 | 2.34877 | 21.3926 |
| NCIH2227 | 33.643 | 0.643658 |
| NCIH510 | 14.2654 | 1.69638 |
| NCIH2066 | 3.20708 | 32.6372 |
| NCIH1341 | 118.748 | 65.4643 |
| NCIH2029 | 11.3455 | 8.00637 |
| COLO668 | 1.55356 | 3.91477 |
| NCIH1963 | 3.4852 | 4.28216 |
| CORL311 | 12.422 | 0.670214 |
| NCIH2286 | 3.26741 | 0.095202 |
| NCIH1339 | 4.11604 | 0.062809 |
| NCIH2081 | 4.00195 | 9.43307 |
| NCIH1092 | 2.55472 | 42.1812 |
| NCIH1105 | 0.969034 | 10.6492 |
| NCIH1436 | 9.58561 | 20.4368 |
| NCIH1876 | 4.73258 | 8.91245 |
| CORL279 | 4.39198 | 12.8149 |
| NCIH1048 | 7.03995 | 2.05658 |
| NCIH1618 | 4.97384 | 12.481 |
| NCIH1930 | 5.39773 | 2.89012 |
| NCIH1836 | 0.213956 | 2.55145 |
| NCIH524 | 118.422 | 4.11126 |
| NCIH209 | 10.1566 | 0.374816 |
| NCIH196 | 3.71276 | 1.08652 |
| NCIH889 | 0.746301 | 13.2175 |
| NCIH211 | 1.46627 | 1.19617 |
| NCIH2171 | 14.1172 | 4.4183 |
| NCIH146 | 5.27795 | 0.0376766 |
| NCIH526 | 4.01984 | 5.69532 |
| NCIH2196 | 6.7172 | 0.618396 |
| NCIH446 | 0.5021 | 45.4482 |
| SCLC21H | 166.317 | 6.37546 |
| NCIH841 | 4.92433 | 0.0444249 |

Figure 2 Quantify transcript levels of ASS1 and SESN3 across CCLE SCLC lines via GEO database (Accession: GSE193455).
